# Choosing a substrate for the ion irradiation of two-dimensional materials

**DOI:** 10.3762/bjnano.10.54

**Published:** 2019-02-22

**Authors:** Egor A Kolesov

**Affiliations:** 1 Belarusian State University, 4 Nezavisimosti Av., 220030 Minsk, Belarus

**Keywords:** 2D materials, defects, hot electrons, ion irradiation, recoils, sputtering, substrate

## Abstract

This study is dedicated to the common problem of how to choose a suitable substrate for ion irradiation of two-dimensional materials in order to achieve specific roles of certain defect formation mechanisms. The estimations include Monte Carlo simulations for He, Ar, Xe, C, N and Si ions, performed in the incident ion energy range from 100 eV to 250 MeV. Cu, SiO_2_, SiC and Al_2_O_3_ substrates were analyzed. The considered substrate-related defect formation mechanisms are sputtering, recoil atoms reaching the interface with a non-zero energy, and generation of hot electrons in close proximity of the interface. Additionally, the implantation of sputtered substrate atoms into the 2D material lattice is analyzed. This work is useful both for fundamental studies of irradiation of two-dimensional materials and as a practical guide on choosing the conditions necessary to obtain certain parameters of irradiated materials.

## Introduction

Ion irradiation of two-dimensional (2D) materials is a versatile and convenient tool for modifying the material structure through a controlled induction of lattice defects, cutting or atom implantation [1–3]. This method is useful for engineering of the optical, electric and catalytic properties of monolayers [1–3].

For technical simplicity, ion irradiation of 2D materials is usually carried out on substrates [1]. However, the substrate choice is known to play a significant (and sometimes crucial) role in the irradiation process [1–7]. On one hand, it can increase the stability of a monolayer under irradiation, leading to reduction of a resultant defect yield [3]; on the other, it can participate in defect formation in the 2D material through energy transfer from sputtered substrate atoms moving through the monolayer [1,4]. When the energy is suitable, these atoms can become embedded into the 2D material crystal lattice, leading to a peculiar doping effect, as it was shown for graphene in [1]. Besides, one cannot exclude participation of displaced recoil atoms [8] that reach the interface but remain within the substrate. Moreover, since there is a charge transfer between the substrate and the monolayer [9–12], the generation of hot electrons in the substrate within the close proximity of the interface can lead to a more intensive electronically stimulated surface atom desorption [13–14], which already occurs directly in a 2D material under ion irradiation [13,15].

In [16] it was shown by Raman spectroscopy and Transport of Ions in Matter (TRIM) simulations using 160 MeV Xe ions for the irradiation of graphene on Cu, SiO_2_/Si and glass leads to negligible overall participation of substrate sputtering and a stronger (but small) role of the substrate recoils. Besides, it was noted that hot electrons generated in the substrate in the vicinity of the interface can possibly lead to introduction of additional defects in the monolayer.

As reported in [1–3,5–8,16], all these effects are generally known in the experimental literature for a specific irradiation energy, substrate and ion type, while simulation studies focus more on defect formation energies and probabilities. At the same time, comparing the expected extent of each mechanism can allow not only the exclusion of unwanted effects, but also the use of them for a more controllable engineering of the process, in order to obtain a desired result through a simple method of choosing the substrate/ion combination.

Due to their nature, 2D materials tend to absorb a small fraction of ion energy in the keV–MeV range [15]. This allows simulations of the bulk substrate effects to be performed independent of the monolayer type. Although not directly applicable to calculating the defect density in irradiated 2D materials, TRIM code allows one to perform massive statistical calculations of damage to bulk targets, such as substrates. Thus, it allows the relative roles of various substrate-related mechanisms of defect formation to be determined in the irradiated 2D materials.

The purpose of the present study is to systematically compare the intensity of defect formation in 2D materials through substrate sputtering, substrate recoils reaching the interface, and generated hot electrons, in order to make it possible to choose the irradiation conditions while taking the substrate effects into account. The analysis was performed for the most common ions used for monolayer irradiation: He, Ar, Xe, C, N and Si; the chosen substrates include Cu, SiO_2_, SiC and Al_2_O_3_. Copper is a widely available metal and is traditionally used as a substrate in 2D material science, initially recognized due to its catalytic effect in the CVD synthesis of graphene. SiO_2_, the most common material for supporting monolayers (usually in the SiO_2_/Si alignment), is in turn a typical dielectric, mostly referred to as introducing a comparatively small effect on the properties of 2D materials. For the uniformity of the study, a classical semiconducting material SiC was added as another commonly used substrate. Al_2_O_3_ is also an insulator that is becoming a more interesting material to support monolayers given that is has a small effect on their properties; besides, comparing it to SiO_2_ is potentially useful since dielectric substrates are highly essential for nanoelectronic applications.

## Results and Discussion

For each simulation a set of output values was obtained describing damage to the target substrate implemented through different processes. Among them, substrate sputtering can be on one hand effectively regarded as a secondary irradiation of the adsorbed two-dimensional material in accordance with a common practice [4]; on the other hand, the substrate recoils reaching the interface may be considered a source of energy transferred to the monolayer while remaining within the substrate [8,16]. Both of these fundamentally different interaction events can lead to introduction of defects to the overlaying material, if the energy is sufficient. It is important to underline that in order to make the results on these mechanisms comparable, they were normalized to a single form of “total ion energy transferred to a process” (eV/ion), which is an indicative parameter for both of them. This representation, utilized below, enables one to take into account sputtering yield/amount of recoils and sputtered atom energy/recoil energy in a single value, and therefore allows the degree to which each phenomenon takes place in relation to another to be established. However, it does not directly show the energy of a single sputtered atom/recoil, thus these values are indicated in the text when describing the key moments of the dependencies.

**Figure 1:**
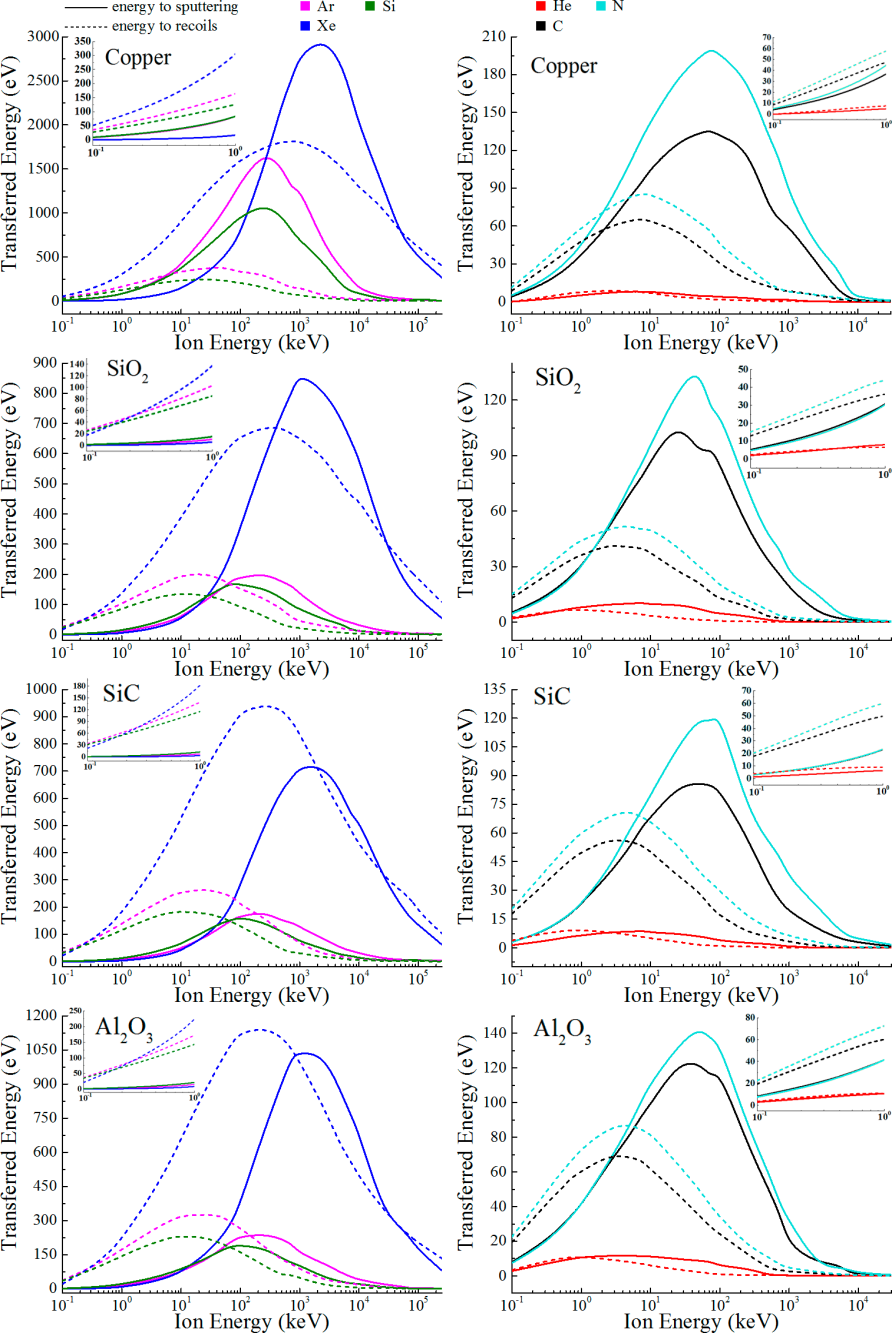
Energy transferred to substrate sputtering (solid lines) or substrate recoils reaching the interface while remaining within the substrate (dotted lines), as a function of the incident ion energy for Cu, SiO_2_, SiC and Al_2_O_3_ substrates and He, Ar, Xe, C, N and Si ions.

Figure 1 presents the dependency of the energy transferred to substrate sputtering or substrate recoils reaching the interface on the energy of the incident ion for Cu, SiO_2_, SiC and Al_2_O_3_ substrates and He, Ar, Xe, C, N and Si ions. For better clarity, the curves for lighter ions (He, C, N) and heavier ions (Ar, Xe, Si) are plotted separately, since for the latter, the maximum values of transferred energy are up to an order of magnitude larger. The insets in Figure 1 show details of the low-energy region (10^−1^–10^0^ keV) of the dependencies presented.

As the incident ion energy increases, the energy transferred to both substrate sputtering and recoils reaching the interface begins to increase notably starting from 10^−1^–10^0^ keV where the ion has enough energy to activate these processes. As seen in the insets, the energy transferred to recoils reaching the interface seems to dominate over sputtering in the low-energy range. However, it should be noted that since there is a large probability of a direct collision interaction within the overlying two-dimensional material in this range (as it was shown for typical and common 2D materials such as graphene and MoS_2_ in [1,4]), the beginning of the increase is expected to be upshifted on the horizontal axis due to ions partially or fully losing their energy before reaching the substrate. Therefore, the details in the insets are shown here to illustrate the uniformity of the presented data rather than for the following predictions which mostly relate to energies above 1 keV. At the same time, the domination of interactions within a 2D material over those in the substrate quickly softens with further incident ion energy increase, and after 1 keV, the substrate-related defect formation dominates (as is commonly reported in other works, e.g., [1,4]). The plots show a pronounced maximum at 10^1^–10^3^ keV, after which a decrease is observed until practically the entire energy loss of the ion becomes transferred to the target electronic excitations [17].

Depending on the substrate material and incident ion mass, the maximum energy transferred to sputtering ranges from ≈8 eV/ion (He ions in copper) to ≈2990 eV/ion (Xe in copper), which corresponds to an average sputtered atom energy of 22.1 and 320.1 eV/atom, respectively. The former value is already enough to introduce defects into common two-dimensional materials [18–22], most strongly manifesting for 2D transition metal dichalcogenides (TMDs) due to the low displacement threshold barrier [18–19]. The latter is sufficient to create a considerable amount of defects – for example, through initiating a horizontal (in-plane) recoil cascade in a two-dimensional material, which is a chain of successive events of recoil atom energy transfer to other atoms (secondary, tertiary, etc.) [17]. For recoils reaching the interface, the maximum transferred ion energy values are comparable to sputtering, with the most pronounced relative role for SiC and Al_2_O_3_ irradiation by xenon. At the same time, individual recoil energies corresponding to curve maxima have average values from 51.7 (oxygen recoils, He ions in SiO_2_) to 204.8 (Xe in copper) eV/atom, with the former value being an order of magnitude greater than that needed to damage 2D TMDs [18–19] and more than two times greater than the amount required to introduce defects in graphene [20–22]. The values given above indicate that the substrate can participate strongly in defect formation in two-dimensional materials under ion irradiation with a wide range of ion energies. At the same time, the deliberate use of these phenomena opens up broad prospects in the controllability of the process.

The plots for recoils reaching the interface generally reach a maxima and start to decrease before those for sputtering. At energies greater than 10^4^ keV, they cease to make a significant contribution, except for Xe irradiation, for which the effect is non-negligible until the upper limit of the studied range (250 MeV). For the latter, the point of 160 MeV discussed in detail in [16] shows stronger participation of recoils against the sputtered substrate atoms for all substrates considered.

Small plot inflections in the high-energy range, such as those for copper sputtering at ≈1 × 10^3^ keV (Ar ions) or for SiC sputtering at ≈1 × 10^4^ keV (Xe ions), are related to the fact that at these ion energies, the number of recoil atoms per incident ion is already decreasing, while their average energy is still growing. Other similar changes in the curvature (second derivative) are also explained by the fact that the plotted quantities are mathematically the product of the process yield with the average atom energy. As the energy of the incident ion increases, these parameters increase/decrease with different rates, which leads to an uneven change in the plot curvature. For compound targets, this effect occurs separately for both atom types, leading to a more complex structure of the curves.

As shown in Figure 1, the maxima for several compound target curves split into two components such as those for C ions in SiO_2_ or N in SiC. This is connected to different yield-energy product maxima for each atom type of the compound target – in the mentioned example of C ions in SiO_2_, the first component at ≈2.5 × 10^1^ keV on the horizontal axis corresponds to prevailing participation of sputtered oxygen atoms, while the second one at ≈7.5 × 10^1^ keV indicates the greater role of silicon atoms. In a similar case of Al_2_O_3_ irradiation with carbon ions, components at ≈2.5 × 10^1^ keV and ≈1 × 10^2^ keV show maximum participation of sputtered oxygen and aluminum, respectively. The “unsplit” curves for compound targets indicate that for a given pair of components, the yield-energy product maxima have close values, and therefore the curve components merge.

In order to introduce defects into a two-dimensional material, sputtered substrate atoms and recoils reaching the interface need to have enough energy to pass the displacement threshold barrier which shows values from about 4 eV for chalcogen atoms in WS_2_ [19] to almost 32 eV for molybdenum in MoS_2_ [18] (this range includes values of ≈5–7 eV for S and Se in 2D TMDs [18–19] and ≈22–23 eV in graphene [20–22]). However, in [1] it was shown that, except for participating in the defect formation in adsorbed monolayers, the substrate can also increase the displacement threshold through creating a barrier for displaced atoms of the two-dimensional material, effectively making them “bounce back” after being displaced. For example, the threshold for graphene supported by a common SiO_2_ substrate increases to 68–196 eV [1], depending on the carbon atom position relative to the substrate oxygen site. Considering the fact that the energy transferred to defect formation in a two-dimensional material upon irradiation with helium is close to the threshold, an important conclusion can be drawn that He ions are the most preferred when it is required to irradiate supported monolayers desirably avoiding the influence of the substrate (generation of hot electrons in the substrate should also be taken into account here, as discussed later).

A basic type of defect produced during 2D material irradiation is a vacancy (for 2D TMDs, the most probable vacancy generation event involves chalcogen atom removal) [23]. If the fluence is enough, multiple generated vacancies can subsequently merge through the migration processes [15,24], leading to a creation of more complex defects. Additionally, the incident ion can become embedded into the 2D material crystal lattice, leading to ion implantation (doping) [25]. For the latter to occur, the ion should have a considerably low energy – of about 20–200 eV, with the most effective implantation occurring at 25–75 eV [1,25–29]. The process peaks in the lower part of this region for 2D TMDs and in the higher regions for graphene. Outside this range, the probability of a direct substitution decreases, although it does not fully vanish [1]. This value increases for supported monolayers since the presence of the substrate can lead to ion backscattering [1]. Simultaneously, the sputtered substrate atoms can receive energy optimal for the implantation as well, leading to doping of the 2D material with the substrate atoms at much greater incident ion energies (for example, 5 keV Si into graphene on SiO_2_ [1]; similar is naturally expected for 2D TMDs). Table 1 presents simulation results for sputtered substrate atom doping, showing the range of incident ion energy in which sputtered substrate atoms have the energy most suitable for the implantation into common 2D materials (25–75 eV/atom [1,25–29]). At the same time, in order to get the information on how actively this process is taking place considering the yield, one should refer to the solid curves for sputtering in Figure 1 within the range specified in Table 1. The results presented in Table 1 are in agreement with molecular dynamics simulations on the subject [1].

**Table 1:** Incident ion energy ranges (in keV) which correspond to the most effective sputtered substrate atom implantation into various 2D materials, i.e., sputtered atoms have energy in the 25–75 eV/atom range.

Substrate		He	Ar	Xe	C	N	Si

Cu		8–85	2–18	21–159	3–28	3–25	2–16
SiO_2_	Si	1–7	5–39	15–103	1–4	1–4	3–18
	O	3–19	18–228	44–347	3–21	3–31	11–224
SiC	Si	1–10	6–55	20–142	1–6	1–6	3–24
	C	1–6	3–37	9–85	1–9	1–12	2–26
Al_2_O_3_	Al	2–20	12–117	31–257	1–11	1–13	6–56
	O	3–25	20–235	48–345	2–23	3–31	12–142

It should be noted that the sputtered substrate atoms can still have energy within the 25–75 eV/atom range at greater incident ion energies of above 10^3^ keV/ion as well, when the energy transferred to sputtering already decreases; however, the implantation in this case can be expected to be negligible due to a very small yield, since most of the incident ion energy loss is transferred to the substrate electronic subsystem [15,17]. Besides, it is important to understand that although the sputtered substrate atom implantation can be negligible, it still does not fully vanish in the whole conventional incident ion energy range. This could be due, for example, to possible events of the sputtered atom giving most of its energy to a 2D material atom during a collision.

The results presented above phenomenologically and qualitatively agree with other works published on the subject for graphene or 2D TMDs [1–3,6,30] and show the same energy regions of active substrate participation in the defect formation in 2D materials, as well as a similar scale and energy-dependent dynamics of the effect. However, the most useful comparison can be performed with the results given in a recent work [31] for graphene, where it underwent ion irradiation both when free-standing and supported with silicon. In order to compare the simulation with the experiment, the results for irradiation with 30 keV He ions were used [31]. The simulation gives us a silicon sputtering yield of 3.73 × 10^−2^ with the average energy of about 164 eV/atom. According to defect yield/stopping power dependencies from [7], this energy corresponds to a defect yield of 5.65 × 10^−3^. For a fluence of 10^15^ cm^−2^, an estimated difference of defect density was obtained for free-standing and supported graphene of 2.1 × 10^11^ cm^−2^, with the experimental value being about 4.6 × 10^11^ cm^−2^ [31]. Considering that this estimation takes into account only damage from the substrate sputtering (not substrate recoils reaching the interface or hot electrons generated in the vicinity of the interface), this is a very reasonable result.

**Figure 2:**
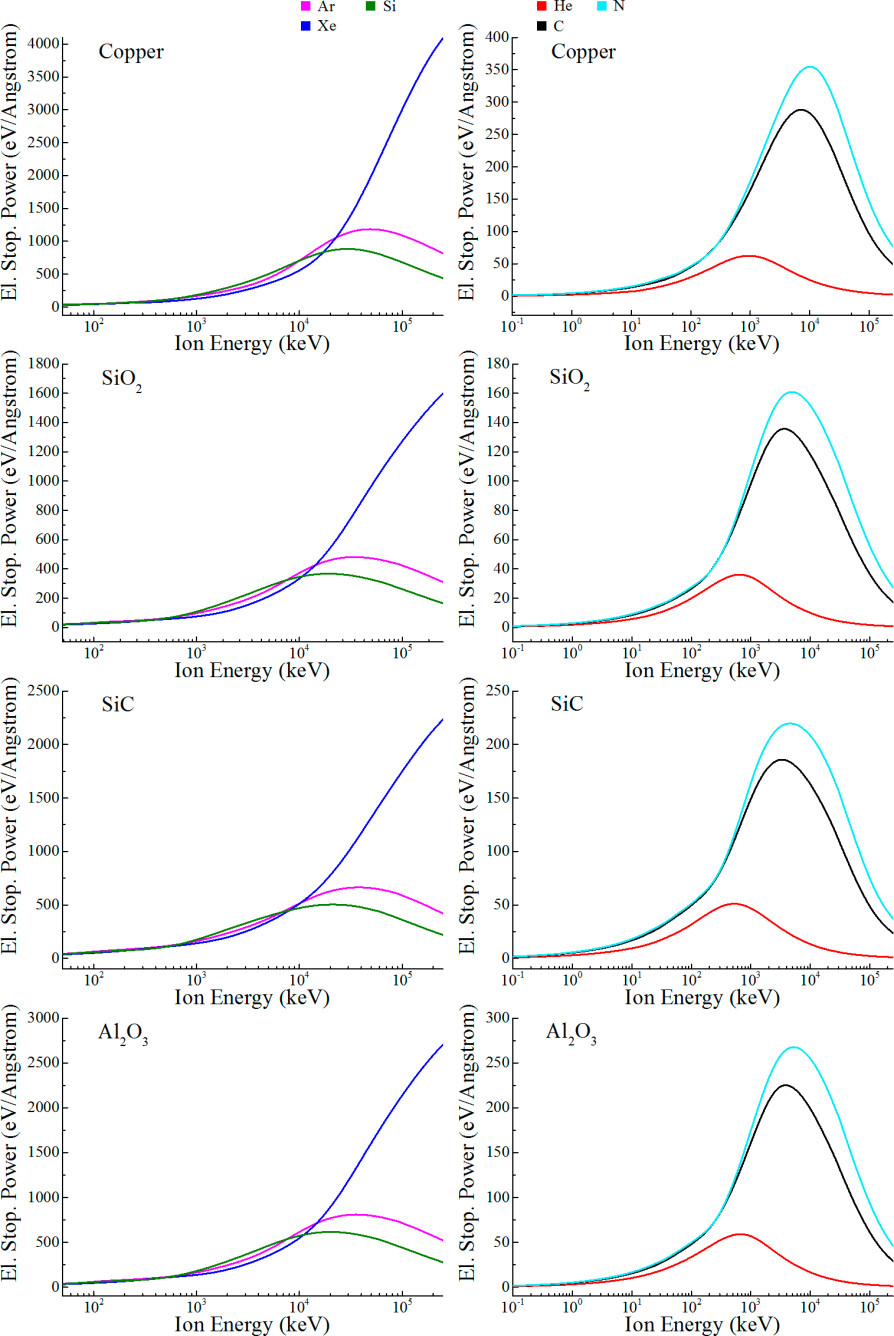
Electronic stopping power dependence on the incident ion energy for Cu, SiO_2_, SiC and Al_2_O_3_ substrates and He, Ar, Xe, C, N and Si ions.

Figure 2 demonstrates dependencies of the energy transferred to the target electronic subsystem (electronic stopping power [17]) on the incident ion energy for Cu, SiO_2_, SiC and Al_2_O_3_ substrates and He, Ar, Xe, C, N and Si ions. The obtained curve forms are typical for the description of inelastic collisions of a penetrating ion with the target electrons, resulting in electron excitations and atom ionizations [17].

For all ions except xenon, the pronounced maxima are observed at the incident ion energy of ≈5 × 10^2^–5 × 10^4^ keV reaching stopping power values of up to 1185 eV/Å (Ar ions in copper). After the maxima, the electronic stopping power decreases long after the nuclear stopping has become negligible [17], indicating the decrease of defect-productive interactions. For Xe irradiation, the maxima are clearly reached at incident ion energies beyond the studied range, with the point of 160 MeV showing a significant amount of energy transferred to substrate electrons as it was assumed in [16]. The overall most pronounced effect is naturally observed for the copper substrate, with the results for Al_2_O_3_ being remarkably close.

While inelastic collisions with the electronic sea naturally do not change the ion trajectory, the maxima in Figure 2 correspond to the strongest relative contribution of this mechanism to the total stopping cross section. At large incident ion energies, more than 99% of the ion energy loss can be attributed to the electronic stopping. As its role increases with the ion energy, massive atom ionization occurs, leading to the generation of hot electrons in the target media. When the distance to the 2D material is smaller than the thermalization length of these high-energy particles (i.e., the hot electrons are generated in the substrate in the vicinity of the interface), they can contribute to the defect formation by electronically stimulated surface atom desorption in 2D material through charge transfer effects [9–12,16]. It is important to note that the electronically stimulated surface atom desorption is a mechanism that basically occurs in unsupported 2D materials under ion irradiation [13]. It principally relates to any non-central collision event with a reduced direct kinetic energy transfer [13], but in practice, it will be most effective for incident ion energy regions in which the electronic energy loss dominates (for example, MeV light ion irradiation or 10^1^–10^2^ MeV heavy ion irradiation in unsupported graphene or 2D TMDs). Substrate hot electrons generated in the vicinity of the interface will intensify this mechanism as an additional charge source, leading to a vacancy concentration increase in the irradiated material as long as the threshold for a vacancy formation by electrons is overcome (for example, ≈80 eV for graphene [13]).

Taking the hot electron mean free path as the minimal requirement of distance to the interface, one can easily estimate the amount of energy transferred to substrate electrons in the vicinity of the interface by an incident ion. For the hot electron mean free path dependencies given in [32], we obtain about 114, 885 and 5328 eV/ion for a copper substrate under irradiation by 5 × 10^2^, 5 × 10^3^ and 5 × 10^4^ keV Ar ions (the third ion energy corresponds to the dependency maximum in Figure 2). For SiO_2_ substrates (mean free path dependencies from [33]), the corresponding values are 380, 1277 and 2835 eV/ion. As shown, even at a comparatively low ion energy of ≈10^2^ keV, substrate electrons can obtain enough energy to overcome a vacancy formation threshold for 2D TMDs or graphene.

It should be noted that since the hot electron thermalization length is generally greater than its mean free path, the distance over which the electrons can participate in defect formation will be in practice greater as well. Correspondingly, this further increases the amount of energy transferred to defect formation in the 2D material that can be expected in practice. This demonstrated participation of the substrate hot electrons in the defect formation in monolayers is in accordance with the previously reported experiments [34]. The effect is expected to be more pronounced for semiconducting 2D materials, such as MoS_2_ and other 2D TMDs, where the electronic energy dissipation is less rapid than, for example, in graphene [34]. The simple logic demonstrated in the discussion above can be utilized to calculate the amount of substrate hot electron energy that can be transferred to a monolayer for a specific case (using the dependencies presented in Figure 2) as well as hot electron mean free path or thermalization length (depending on the accuracy required) that can be easily found elsewhere due to the wide availability of this data.

According to the presented results, substrate electrons introduce the weakest effect for SiO_2_ and the strongest one for copper, among the substrates analyzed. The maximum energy lost due to electronic stopping varies from 36 eV/Å for He ions to 4090 eV/Å for Xe irradiation. The estimated numbers show the importance of taking into account the generation of substrate hot electrons when choosing substrate material, ion type and energy for the irradiation of supported 2D materials.

Given the results presented, extending the above discussions for other, less common ion or substrate types, as well as other incident ion angles or ionic charge states, can be considered a promising direction in the search for the best combinations of conditions that are optimal for a desired extent and type of 2D material structural modification by ion irradiation.

## Conclusion

Monte Carlo simulations were performed to estimate the role of Cu, SiO_2_, SiC and Al_2_O_3_ substrates in the defect formation in 2D materials under irradiation with He, Ar, Xe, C, N and Si ions in the incident ion energy range from 100 eV to 250 MeV. The effects of substrate sputtering, substrate recoils reaching the interface with a non-zero energy, and hot electrons generated in the substrate in a close proximity of the interface were shown to be non-negligible, considering defect formation energies in common 2D materials. For all ions except Xe, the most significant role for sputtering was found at 10^1^–10^3^ keV, for recoils at 10^0^–10^2^ keV, and for hot electrons at 10^3^–10^5^ keV. For Xe, the most active participation of both sputtering and recoils was at 10^2^–10^4^ keV, while the role of hot electrons gradually increased in the whole studied energy range. He ions were shown to be the most preferred when supported monolayers are to be irradiated, desirably avoiding the influence of substrate sputtering and recoils. SiO_2_ was the substrate with the smallest effect of the analyzed mechanisms. In addition, incident ion energy ranges were presented in which the implantation of sputtered substrate atoms into the 2D material crystal lattice effectively occurs. The present work is useful both for providing a fundamental insight into the relative roles of various substrate-related defect formation mechanisms in two-dimensional materials and as a convenient way to choose the irradiation conditions necessary to obtain certain parameters of irradiated materials during nanoelectronic device engineering.

## Modelling

The calculations were performed using a binary collision Monte Carlo approach implemented in TRIM code [35]. TRIM code uses a quantum-mechanical collision treatment that considers screened Coulomb interaction between the incident ions with an effective charge and the target atoms, including exchange and correlation interactions for the overlapping electron shells, as well as creation of electronic excitations or plasmons inside the target [35].

TRIM treats a target as an amorphous matrix with a homogenous mass distribution and calculates collision impact regardless of collision density. Thus, it is very important to underline that when applied in a straightforward manner, this method fits bulk materials only and is not applicable for direct modelling of defect formation in the freestanding 2D material [15]. However, the statistical Monte Carlo approach is very useful when it comes to substrate-related effects [3–4,7,16]. It allows one to determine number and energy of sputtered substrate atoms or substrate recoils reaching the 2D material–substrate interface with a non-zero energy, as well as information on hot electrons generated in the substrate in the vicinity of the interface. Consequently, these results can then be used to estimate an impact on a 2D material, utilizing the known dependencies for the defect formation in freestanding 2D materials such as that in [7]. Since the substrate is a bulk material, its displaced atoms can reach the surface from a considerable depth, depending on the incident ion type and energy. The statistical approach of TRIM allows such recoils to be taken into account without having to utilize significant computing power, which in turn makes it possible to build resultant dependencies over a wide incident ion energy range for a variety of ions and substrates.

In the present study, the monolayer collision mode (in which every collision is calculated without any approximations) with 100,000 incident He^+^, Ar^+^, Xe^+^, C^+^, N^+^ or Si^+^ ions was utilized for Cu, SiO_2_, SiC or Al_2_O_3_ substrates with a thickness of 300 Å for a 0° angle (i.e., normal incidence). The parameters describing the substrate material or ion were assigned within the framework of a built-in TRIM database. During the calculation, the sputtering of the substrates was taken into account through the two basic TRIM output parameters for the monolayer collision/surface sputtering mode: average sputtering yield (atoms/ion) and average resultant target atom energy (eV/atom). Multiplying these parameters obviously gives the ion energy transferred to sputtering. For compound targets, the total energy to sputtering was summarized. The data on recoils reaching the 2D material–substrate interface with a non-zero energy while remaining within the substrate was obtained using two TRIM calculation output distributions: the recoil distribution, which shows spatial recoil positions and therefore can be utilized to find the average number of recoils reaching the interface per incident ion, and the spatial plots of ion energy transferred to recoils. Using these parameters allows one to compare the two mechanisms on a single scale in terms of both energy and yield.

## References

[1] Zhao S, Xue J, Wang Y, Yan S (2012). Nanotechnology.

[2] Madauß L, Ochedowski O, Lebius H, Ban-d’Etat B, Naylor C H, Johnson A T C, Kotakoski J, Schleberger M (2016). 2D Mater.

[3] Mathew S, Chan T K, Zhan D, Gopinadhan K, Barman A-R, Breese M B H, Dhar S, Shen Z X, Venkatesan T, Thong J T L (2011). Carbon.

[4] Kretschmer S, Maslov M, Ghaderzadeh S, Ghorbani-Asl M, Hlawacek G, Krasheninnikov A V (2018). ACS Appl Mater Interfaces.

[5] Compagnini G, Giannazzo F, Sonde S, Raineri V, Rimini E (2009). Carbon.

[6] Mathew S, Chan T K, Zhan D, Gopinadhan K, Roy Barman A, Breese M B H, Dhar S, Shen Z X, Venkatesan T, Thong J T L (2011). J Appl Phys.

[7] Li W, Wang X, Zhang X, Zhao S, Duan H, Xue J (2015). Sci Rep.

[8] Herbig C, Åhlgren E H, Jolie W, Busse C, Kotakoski J, Krasheninnikov A V, Michely T (2014). ACS Nano.

[9] Davydov S Y (2011). Tech Phys Lett.

[10] Li Y, Qi Z, Liu M, Wang Y, Cheng X, Zhang G, Sheng L (2014). Nanoscale.

[11] Kong L, Bjelkevig C, Gaddam S, Zhou M, Lee Y H, Han G H, Jeong H K, Wu N, Zhang Z, Xiao J (2010). J Phys Chem C.

[12] Romero H E, Shen N, Joshi P, Gutierrez H R, Tadigadapa S A, Sofo J O, Eklund P C (2008). ACS Nano.

[13] Krasheninnikov A V, Miyamoto Y, Tománek D (2007). Phys Rev Lett.

[14] Lenner M, Kaplan A, Huchon C, Palmer R E (2009). Phys Rev B.

[15] Krasheninnikov A V, Nordlund K (2010). J Appl Phys.

[16] Kolesov E A, Tivanov M S, Korolik O V, Apel P Y, Skuratov V A, Saad A, Komissarov I V (2018). J Mater Sci: Mater Electron.

[17] Nastasi M, Mayer J, Hirvonen J K (1996). Ion–solid Interactions.

[18] Ghorbani-Asl M, Kretschmer S, Spearot D E, Krasheninnikov A V (2017). 2D Mater.

[19] Komsa H-P, Kotakoski J, Kurasch S, Lehtinen O, Kaiser U, Krasheninnikov A V (2012). Phys Rev Lett.

[20] Merrill A, Cress C D, Rossi J E, Cox N D, Landi B J (2015). Phys Rev B.

[21] Skowron S T, Lebedeva I V, Popov A M, Bichoutskaia E (2015). Chem Soc Rev.

[22] McKenna A J, Trevethan T, Latham C D, Young P J, Heggie M I (2016). Carbon.

[23] Lin Z, Carvalho B R, Kahn E, Lv R, Rao R, Terrones H, Pimenta M A, Terrones M (2016). 2D Mater.

[24] Wadey J D, Markevich A, Robertson A, Warner J, Kirkland A, Besley E (2016). Chem Phys Lett.

[25] Bangert U, Pierce W, Kepaptsoglou D M, Ramasse Q, Zan R, Gass M H, Van den Berg J A, Boothroyd C B, Amani J, Hofsäss H (2013). Nano Lett.

[26] Li W, Xue J (2015). RSC Adv.

[27] Cress C D, Schmucker S W, Friedman A L, Dev P, Culbertson J C, Lyding J W, Robinson J T (2016). ACS Nano.

[28] Murray R, Haynes K, Zhao X, Perry S, Hatem C, Jones K (2016). ECS J Solid State Sci Technol.

[29] Åhlgren E H, Kotakoski J, Krasheninnikov A V (2011). Phys Rev B.

[30] Agius Anastasi A, Valsesia A, Colpo P, Borg M K, Cassar G (2018). Diamond Relat Mater.

[31] Maguire P, Fox D S, Zhou Y, Wang Q, O'Brien M, Jadwiszczak J, Cullen C P, McManus J, Bateman S, McEvoy N (2018). Phys Rev B.

[32] Da B, Shinotsuka H, Yoshikawa H, Ding Z J, Tanuma S (2014). Phys Rev Lett.

[33] Garmash V I, Djuzhev N A, Kirilenko E P, Makhiboroda M A, Migunov D M (2016). J Surf Invest: X-Ray, Synchrotron Neutron Tech.

[34] Ochedowski O, Marinov K, Wilbs G, Keller G, Scheuschner N, Severin D, Bender M, Maultzsch J, Tegude F J, Schleberger M (2013). J Appl Phys.

[35] Ziegler J F, Ziegler M D, Biersack J P (2010). Nucl Instrum Methods Phys Res, Sect B.

